# Age-related shifts in dietary and oviposition preferences and steep costs of modest parental ageing in *Drosophila melanogaster*

**DOI:** 10.1098/rspb.2026.1983

**Published:** 2026-11-11

**Authors:** Oonagh Barker, Edward R. Ivimey-Cook, Wilfried Haerty, Alexei A. Maklakov, Tracey Chapman

**Affiliations:** ^1^ School of Biological Sciences, University of East Anglia, Norwich, UK; ^2^ Earlham Institute, Norwich Research Park, Norwich, UK

**Keywords:** ageing, nutrition, diet choice, parental effects, transgenerational effects, life history

## Abstract

Nutritional requirements vary across sexes and lifetimes and are predicted to influence dietary preferences. Here, we tested the hypothesis that diet preferences vary adaptively with sex, age and life stage. We used the fruit fly *Drosophila melanogaster* to take independent snapshots of choice for three protein : carbohydrate (P : C) diets in both sexes of young (5 d), mid-aged (15 d) or old (25 d) flies, while simultaneously recording oviposition site choice. We then tested, for mothers of each age, the diet preferences, development and survival on each diet of their larval offspring. Diet choice was sex- and age-specific. Males consistently preferred high carbohydrate, and females high protein (and these preferences became stronger with age). By contrast, oviposition choice was for balanced (1 : 1 P : C) and high carbohydrate diets in young and old females, respectively. Larvae of old, but not young, parents also preferred balanced diets. Young-parent larvae developed faster on balanced diets, with survival differences (highest with high carbohydrate) evident only in mid-aged-parent larvae. Unexpectedly, mid-aged-parent larvae had slower development and higher preadult mortality than young-parent larvae. Thus, diet choices may not be optimized across parents and offspring, and we identify novel effects of modest increases in parental age and offspring diets on offspring fitness.

## 1. Introduction

The evolution of sex- and age-specific dietary preferences confers the opportunity for individuals to selectively forage for optimal combinations of nutrients to maximize their reproductive potential [1–3]. In insects, males and females often maximize their reproductive output at different intakes of carbohydrate (C) relative to protein (P), leading to contrasting dietary preferences that can underpin sex-specific variation in lifespan [4–8]. Sex- and life-stage-specific dietary responses have been comprehensively described in the fruit fly *Drosophila melanogaster*, which has long been recognized as a valuable model for biogerontology and for studying the interplay between diet and ageing in particular [9–12].

In male *D. melanogaster*, both longevity and reproduction are maximized on high C diets [5,6]. By contrast, in females, while lifespan is also maximized on high C, reproductive output is maximized on diets containing high P [5,6,9,13–15]. There are also sex-specific effects in dietary responses to mating. Unmated individuals of both sexes prefer to feed along a P : C ratio of 1 : 4 [5]. However, female, but not male, dietary intake changes significantly after mating ([16–21], but see [22]). Following mating, females ingest significantly more protein [16–19,21]. This dietary switch occurs as part of a suite of post-mating changes and is mediated by a specific component of the male ejaculate—the ‘sex peptide’ [16,18,19,21].

The evolution of variable dietary preferences allows individuals to potentially select the optimal combination of nutrients according to age and life stage. For example, in females, lifetime reproductive output could potentially be maximized by adaptively adjusting P and C intake to balance longevity and egg productivity according to each age. As high C maximizes both lifespan and reproduction in males [5,6], dietary preference for high C/low P is expected to be sustained throughout a male's lifetime. Senescence may impair the ability of organisms to adaptively optimize diet selection by affecting the pathways through which nutrients are sensed and metabolized [23]. Dietary optimization throughout life may also be constrained because older flies of both sexes eat less and are less able to increase food intake to compensate for low dietary protein, with females more adversely affected in this regard than males [24]. This process is known as ‘protein leveraging’ and has been explored as a potential driver of obesity in humans [25]. Ultimately, sex differences in nutrient sensing over an individual's lifetime probably interact with sex-specific reproductive senescence [26,27].

Diet choice affects not only the lifespan and reproductive output of the focal, but also future generations. In species such as fruit flies that selectively lay eggs on larval food substrates, maternal oviposition choice will affect key fitness-related traits in offspring. Oviposition site selection is even considered a rudimentary form of parental care [28,29]. Female *D. melanogaster* are reported to assess the concentration and chemical identity of sweet compounds [30], show sensitivity to mechanosensory properties [31,32] and respond to the microbial environment [33] when selecting oviposition sites. Oviposition site preference can be for low [28,34] or higher P : C ratios [35], and ovipositing females are reported to avoid high sucrose [30,36,37]. Females might be expected to prefer to lay eggs on diets that speed up larval development and increase survival [28,35]. It is also not known how oviposition preferences vary with age. It is possible that females could become more selective with age because egg quality potentially declines, which could place increased importance on compensatory feeding by larvae. Alternatively, older females could show reduced sensitivity to nutritional differences in oviposition sites owing to age-related senescent declines in nutrient sensing. As developmental diet is also an important determinant of early-life adult fecundity [38], changes in oviposition site selection are likely to affect offspring quality. Beyond this direct effect of oviposition site selection on offspring fitness, the developmental diet of parents can have inter- and trans-generational effects [39–43].

To date, the best-known example of inter-generational influence of parental age is known as the ‘Lansing Effect’, in which advanced maternal age is associated with reduced offspring lifespan. Evidence for the Lansing Effect across animal taxa is patchy, with the most consistent effects reported in insects and rotifers [44]. However, much less is known about how parental age effects are filtered through environments experienced by offspring. That such effects are potentially important is suggested by findings in the red flour beetle *Tribolium castaneum*, in which offspring of older parents are less resilient to starvation than offspring of younger parents, despite pupating later at a higher body mass [45]. Investigating how offspring of different parental ages are affected by rearing conditions, such as the nutritional environment, is therefore a potentially overlooked source of life history trait variation.

In the present study, we tested how dietary, and, in adult females, oviposition, preferences changed with life stage, sex and age (supplementary material, figures S1 and S2). The preferences of larvae of females of different ages were then scored. To assess potential fitness effects of observed preferences, we separately measured the effect of each diet on development and preadult survival of the offspring of mothers of differing ages. We scored adult and larval diet and oviposition preferences by using solid, semi-defined diets [46]. The presence of individuals and the number of eggs laid on or in different food patches was used as the measure of preference. This is an indirect proxy for diet preference but is strongly associated with food ingestion [47]. Importantly, this preference method enabled direct cross-comparison of diet choices versus responses between life-stages, traits and sexes.

We measured the diet preferences of independent sets of mated males and females at three different ages (5 d-, 15 d- and 25 d-old), which corresponded to early, mid, and mid-to-late across the reproductive lifespan for females in this species. Groups of mated, single-sex adults were offered a free choice between 3 different P : C diets (1 : 4, 1 : 1 and 4 : 1) in petri dish arenas (supplementary material, figure S3). Simultaneously, we recorded oviposition site preferences of females by counting the number of eggs laid on each diet patch. Dietary preferences of larvae from mothers of all three ages were then tested. Fitness effects of observed choices were then assessed by placing larvae of 5 and 15 d mothers separately on each of the three test diets.

Our hypotheses were that diet preferences would be adaptive according to sex, age and life stage, with beneficial effects on offspring development and survival. We tested the following predictions:

— 1. Males would consistently prefer high carbohydrate (1 : 4) P : C diets, which maximize both reproduction and lifespan ([5,6, p. 200]; supplementary material, figure S1a).
— 2. Females would favour feeding on higher protein (1 : 1 or 4 : 1) diets to support high fecundity [5,48]. We expected this preference for protein to become weaker with age, owing to reproductive senescence (supplementary material, figure S1b).
— 3. Females would lay eggs predominantly on the 1 : 1 patch, which benefits larval development [28,34,35], or, by contrast to feeding patterns, lay on 1 : 4, as reported [28,34]. In either case, we expected the preference to decline with age owing to reproductive senescence.
— 4. Larvae would consistently favour the 1 : 1 patch, which is reported to accelerate development [34] and improve survival and learning performance [28]. Larvae were predicted to avoid the 4 : 1 patch owing to its high, and possibly larval-toxic, levels of protein [49,50].
— 5. Larvae reared directly on balanced 1 : 1 P : C diets (predicted to be preferred for egg-laying by mothers and for feeding in by larvae) would show the highest survival, largest body sizes and fastest development, in comparison with larvae reared on the other two diets.

## 2. Material and methods

### (a) Fly stocks

Experimental flies were sourced from a large, outbred population of wildtype Dahomey *Drosophila melanogaster* [51]. Stocks were maintained on a standard sugar–yeast–agar (SYA) diet (50 g sugar, 100 g brewer's yeast, 15 g agar, 30 ml 10% Nipagin, 3 ml propionic acid, 970 ml water), which represents a P : C ratio of approximately 1 : 2. Experimental flies were maintained throughout in a controlled environment with a 12 : 12 h light : dark cycle at 25°C and 50% humidity. Eggs were collected from population cages on purple grape juice agar plates spread with a small quantity of live yeast paste. Egg collection plates were quartered and placed in 70 ml bottles of SYA to rear the first generation. At 4 d post-eclosion, adults from the first generation were transferred to egg collection cages with access to plates of yeasted purple grape juice agar and allowed to lay overnight. Adults were then removed and egg collection plates incubated for a further 24 h, after which first instar larvae were picked into 7 ml SYA vials at a density of 25 larvae per vial. This ensured that the second generation (used in choice assays) had standardized parental age, and that their offspring (used in larval choice assays and no-choice assays of preadult survival) had standardized grandparental age.

At 2 d post-eclosion, experimental adults for the choice assays were transferred to new vials containing 7 ml SYA media under light CO_2_ sedation. At this time, flies were sorted into mixed-sex groups of 10 males and 10 females per vial, to eliminate any effect of uneven sex ratio. High survivorship was observed under these conditions up to 25 d, which was the oldest age tested, and balanced sex ratios were maintained, with a maximum of 1–2 deaths observed per vial. Experimental adults from this cohort (*N* = 1250) were randomly sampled at 5, 15 and 25 d post-eclosion and divided between choice assays or egg collection cages (supplementary material, figure S2). Media changes in the main mixed-sex groups maintained on SYA were carried out every 3–4 d with the use of light CO_2_ sedation and occurred 2d before each choice assay to standardize time since exposure to CO_2_ across all treatments. Experiments were carried out in a single block.

### (b) Experimental diets

Meridic diets (supplementary material, tables S1–S5) were made following a protocol adapted from [46] in which P and C are provided in the form of casein and sucrose, respectively. Diets were made with P : C ratios of 1 : 4, 1 : 1 and 4 : 1, with a combined macronutrient content (total grams of casein and sucrose) for each diet of 120 g l^−1^.

### (c) Dietary choice assays

In all treatment groups, choice assays were conducted by allowing individuals a free choice between three 2 cm square cut-outs of 1 : 4, 1 : 1 or 4 : 1 media. All assays were conducted within 10 cm diameter plastic petri dishes (supplementary material, figure S3).

### (d) Dietary choice in mated males and females

Adult males and females (*N* = 900 each) were all maintained in mixed-sex groups (10 females + 10 males per vial) all on standard SYA food, up until the point that their diet preferences for 1 : 4, 1 : 1 or 4 : 1 media were tested, at 5, 15 or 25 d of age (supplementary material, figure S2). At each testing day, a random sample of the experimental flies was selected for testing without replacement. Therefore, each group of males or females tested were naive to the test diets, allowing us to test for ageing effects on diet preference responses. For the diet preference testing, separate cohorts of 5, 15 and 25 d post-eclosion adults were transferred separately to 15 choice assay plates per sex and age group in single-sex groups of 10 under light CO_2_ sedation (N total replicates = 90; supplementary material, figure S3a). Following 1 h of acclimatization, plates were observed for eight consecutive intervals, every 30 min, and the number and location of every fly in each plate (i.e. on each food patch, or not on any patch) were counted once at each 30 min time point.

### (e) Oviposition choice

Following the adult dietary choice assay observations above, the test plates (each containing 10 females, *N* = 15 plates per age group, total *N* = 45 plates) were retained for 24 h to allow females to oviposit on their preferred diet patches. After this, plates were frozen at −20°C for at least 4 h to arrest egg development and then defrosted in batches for counting. The sides of each food patch were carefully cut away to a depth of approximately 1 mm and laid flat so that each surface that females could access for egg-laying could be viewed from above in a single image. Photographs of patches containing eggs (*N* = 135) were taken using an Eagle-M microscope with a GXCAm screen. Egg counts were then conducted by eye using a counting tool in ImageJ v.1.54i [52].

### (f) Dietary choice in larvae

Eggs were collected from the mixed-sex adult cohort maintained on SYA food at 5, 15 and 25 d post-eclosion. First instar larvae (*N* = 450) were collected from eggs laid on plates of yeasted purple grape juice agar by females of these respective ages. Larvae were picked from these plates in groups of 10 and deposited at the intersection of three contiguous patches of the 1 : 4, 1 : 1 or 4 : 1 media placed in the centre of a petri dish (supplementary material, figure S3b). Plates were then covered and incubated for 48 h, at which time larvae were manually extracted from media patches and counted (*N* = 15 replicate plates per parental age group, total *N* = 45). A snapshot of dietary preference was then determined by counting the number of larvae present in each of the three diet patches after the 48 h foraging period.

### (g) Offspring development—under ‘no choice’ conditions

Eggs were collected from adult cohorts at 5 and 15 d post-eclosion from the same egg collection plates as those used to source individuals for larval dietary choice assays described above. For each parental age group, five first instar larvae were transferred into 30 vials each of 1 : 4, 1 : 1 and 4 : 1 P : C media (total *N* = 900). Plugs of test media were 1 cm deep and placed on top of 7 ml agar (containing 10 ml Nipagin and 3 ml Propionic acid per litre as preservatives). This ensured that the plugs of test media remained sufficiently moist for the duration of larval development. Vials were monitored once every 2 d until the appearance of pupae, after which vials were monitored daily for eclosed adults until day 14. This cut-off point was chosen as eclosion typically peaks in the stock population after 9–10 d, and so a 14 day cut-off would allow ample time for all viable individuals to eclose. Vials were then frozen at −8°C for subsequent measurement of wing and thorax sizes (supplementary material, figure S4).

### (h) Statistical analyses

Statistical analyses were carried out in R v.4.3.2 (The R Foundation for Statistical Computing, Vienna, Austria, http://www.r-project.org). A table of model type by generation and trait (supplementary material, table S6) and full model outputs (supplementary material, tables S7–S15) are available in the supplementary material. All models were constructed in glmmTMB v.1.1.14 [53]. For all analyses, model fits, zero inflation and dispersion were checked using DHARMa v.0.5.0 [54] and pairwise comparisons with Tukey correction performed using the R package emmeans v.2.0.3 [55]. Observation-level random effects were used to control overdispersion when their inclusion improved model fit [56].

Diet choice in adult males and females, which was measured in the form of snapshot counts of individuals gathered on each patch, or no patch, and repeated at eight time points per assay at each age, was analysed using a negative binomial generalized linear mixed effects model (GLMM). In larvae, where diet choice was measured at a single timepoint with limited zero inflation, better model fit was achieved using a Poisson GLMM. For oviposition choice, we constructed a two-column response matrix of ‘successes’ (an egg laid on a patch) versus failures (eggs laid on other patches) for each replicate. This approach allowed us to fit a beta-binomial distribution to account for the high zero-inflation and overdispersion present in the dataset, which was largely attributable to the expected lower number of eggs laid by older versus younger females. Wing and thorax lengths (supplementary material, §§S1.2 and S1.3) were analysed using Gaussian LMMs.

Development time, measured as the time in days taken by individual offspring to emerge as adults, was first analysed using a mixed effects Cox Proportional Hazards model that was fitted and checked using the R package Survival v.3.8-6 [57]. Owing to non-adherence to proportional hazards, this was followed by a two-part event history analysis that modelled time-to-event (development time) and probability-of-event (the probability that offspring emerged as adults at all). Development time was modelled with a Conway–Maxwell–Poisson distribution, which provided the best model fit given under-dispersion. The 4 : 1 reared group was excluded from the analysis of development time (supplementary material, §S1.1) because there were very few eclosions from 15 d parents on this diet (*N* = 15 of a possible 150), which caused issues with model convergence. Probability of eclosion, which included the 4 : 1 reared group, was analysed using a binomial GLMM.

## 3. Results

### (a) Sex- and age-specific variation in diet choice

Diet selection by adult males and females was determined by a three-way interaction between sex, diet and age (*χ*^2^ = 100.6, d*f* = 6, *p* ≤ 0.0001; supplementary material, table S7). Pairwise comparisons between age groups (supplementary material, table S16) showed striking age and sex differences in how frequently males and females visited diet patches versus no patch at all. 25 d males were less frequently observed on 1 : 1 (*25*–*5 d* estimate = −0.95, *p* < 0.0001; *15*–*25 d* estimate = 1.29, *p* < 0.0001) and 4 : 1 (*25*–*5 d* estimate = −0.38, *p* < 0.01; *15*–*25 d* estimate = 0.40, *p* < 0.01) diet patches, preferring no patch overall (*25*–*5 d* estimate = 0.46, *p* < 0.0001; *15*–*25 d* estimate = 0.64, *p* < 0.0001). However, male preference for the 1 : 4 diet patch did not change with age, consistent with prediction no. 1 (*25*–*5 d* estimate = −0.21, *p* = 0.29; *15*–*5 d* estimate 0.04, *p* = 0.29; *15*–*25 d* estimate = 0.25, *p* = 0.16).

Female preference for diet type varied significantly with age, whereas the overall number of females present on food patches versus no patch remained constant (*25*–*5 d* estimate = 0.06, *p* = 0.39; *15*–*5 d* estimate = −0.02, *p* = 0.88; *15*–*25 d* estimate = −0.09, *p* = 0.17). The 1 : 4 diet patch was chosen more frequently by 15 d females than by 25 d females (*15*–*25 d* estimate = 0.60, *p* < 0.001). The 1 : 1 food patch was favoured more by 15 and 25 d females than by 5 d females (*25*–*5 d* estimate = −0.67, *p* < 0.0001; *15*–*5 d* estimate = −0.44, *p* < 0.001; *15*–*25 d* estimate = 0.23, *p* = 0.18). Similarly, the 4 : 1 food patch was favoured equally by 15 and 25 d females (*15*–*25 d* estimate = −0.01, *p* = 0.99), but less so by 5 d females (*25*–*5 d* estimate = 0.40, *p* = 0.001; *15*–*5 d* estimate = 0.39, *p* = 0.002). These findings contradict prediction no. 2, as females did not reduce occupancy of the P-rich diet patches as they aged, but instead older females were more often present on P-rich patches relative to the youngest group (figure 1).

**Figure 1.**
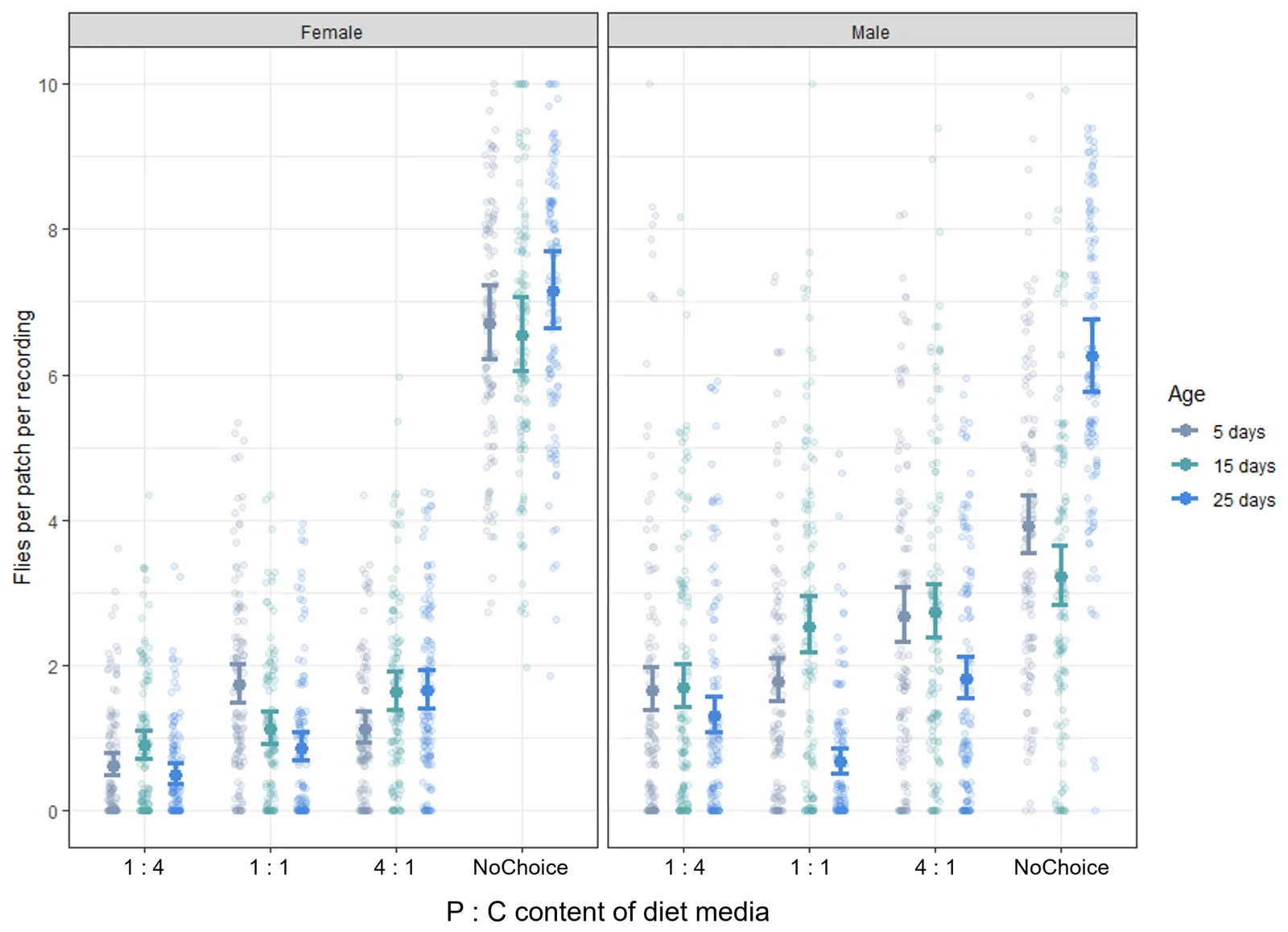
Diet preferences of 5, 15 and 25 d females (left, *N* = 150 per age group) and males (right, *N* = 150 per age group) for 1 : 4, 1 : 1, 4 : 1 (P : C) or no diet patches. Shown are numbers of flies per diet patch per observation (8 × 30 min intervals sampled per replicate, *N* = 15/sex/age group) for each of the diet patches. ‘NoChoice’ refers to individuals not on any diet patch at the time of observation. Points show estimated marginal means and error bars represent 95% asymptotic (Wald-type) confidence intervals calculated in emmeans [55].

### (b) Age-dependent oviposition site selection by females

As for the dietary choice assays reported above, there was a significant interaction between the nutritional content of each diet patch and female age on the proportion of eggs laid per diet patch (*χ*^2^ = 88.0, d*f* = 4, *p* = <0.0001; supplementary material, table S8). Pairwise comparisons (supplementary material, table S17) showed that 25 d females laid proportionately more eggs on the 1 : 4 diet patch (*15*–*25 d* estimate = −1.60, *p* < 0.0001; *25*–*5 d* estimate = 1.76, *p* < 0.0001) and fewer eggs on the 1 : 1 diet patch (*15*–*25 d* estimate = 1.41, *p* < 0.0001; *25*–*5 d* estimate = −1.33, *p* < 0.0001) than did 5 or 15 d females, whereas all age groups laid a consistent, smaller proportion of eggs on the 4 : 1 diet patch (*15*–*25 d* estimate = −0.32, *p* = 0.60; *15*–*5 d* estimate = −0.56, *p* = 0.18; *25*–*15 d* estimate = −0.24, *p* = 0.69). Therefore, prediction no. 3 was partially supported in that oviposition site selection was age-dependent, but the direction of the changes seen was not as predicted (figure 2).

**Figure 2.**
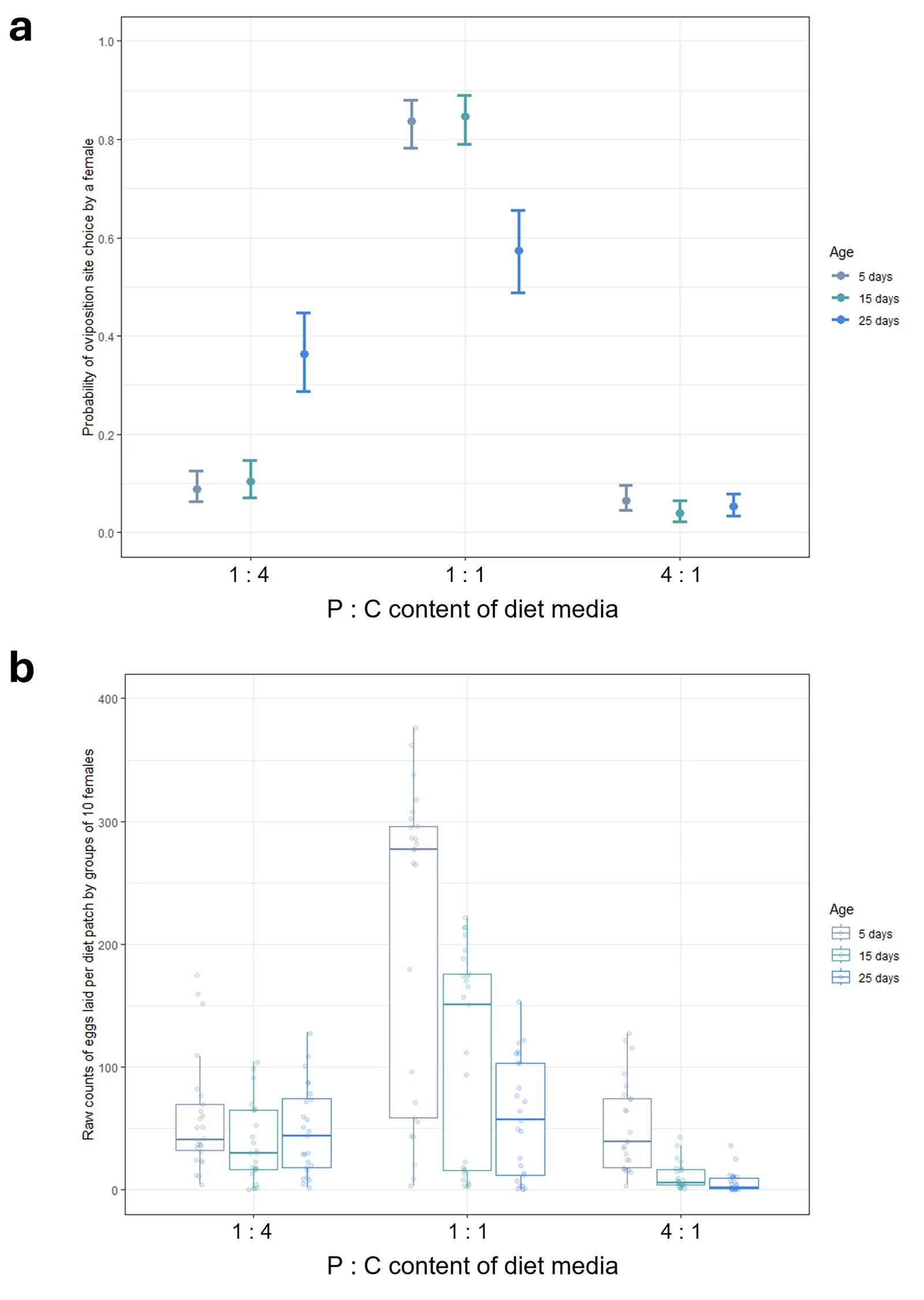
Oviposition site selection by 5, 15 and 25 d females (*N* = 150 individuals per age group. (A) The probability that females from each age group will deposit an egg on the three P : C patch options. Points show estimated marginal mean probabilities of oviposition site choice for each female age group derived from the beta-binomial GLMM. Error bars represent 95% asymptotic (Wald-type) confidence intervals calculated in emmeans [55]. (B) Raw counts of eggs deposited on each patch by groups of 10 females.

### (c) Larvae of only older parents preferred a balanced P : C diet

Nutritional content of the diet patches predicted larval dietary choice (*χ*^2^ = 48.0, d*f* = 2, *p* ≤ 0.0001). Surprisingly, there was a significant interaction between the nutritional content of diet patches and parental age in determining diet choice by larvae (*χ*^2^ = 11.49, d*f* = 4, *p* ≤0.02). Parental age alone had no effect on the total number of larvae recovered (*χ*^2^ = 0.27, d*f* = 2, *p* = 0.87; supplementary material, table S9).

Pairwise comparisons (supplementary material, table S18) showed that the expected preference (prediction no. 4) of larvae for the 1 : 1 P : C diet patch was apparent only at older parental ages. 1 : 1 was favoured above 1 : 4 by larvae of 15 d parents (*1 : 1 – 1 : 4* estimate = 1.41, *p* < 0.0001) and larvae of 25 d parents (*1 : 1 – 1 : 4* estimate = 1.05, *p* < 0.001) but not larvae of 5 d parents (*1 : 1 – 1 : 4* estimate = 0.50, *p* = 0.07). Larvae of 15 d parents also showed a preference for 1 : 1 over 4 : 1 (*1 : 1 – 4 : 1* estimate = 0.85, *p* < 0.001), which was absent in larvae of 5 d or 25 d parents, whereas larvae of 25 d parents showed a preference for 4 : 1 over 1 : 4, which was absent in the 5 d and 15 d parent groups (*1 : 4 – 4 : 1* estimate = 0.83, *p* < 0.001). To summarize, larvae of 5 d parents did not show significant preferences for any food patch; larvae of 15 d parents preferred 1 : 1 over both 1 : 4 and 4 : 1, showing the expected pattern, with no difference in preference between non-preferred 1 : 4 and 4 : 1; and larvae of 25 d parents preferred 1 : 1 or 4 : 1 equally over 1 : 4 (figure 3).

**Figure 3.**
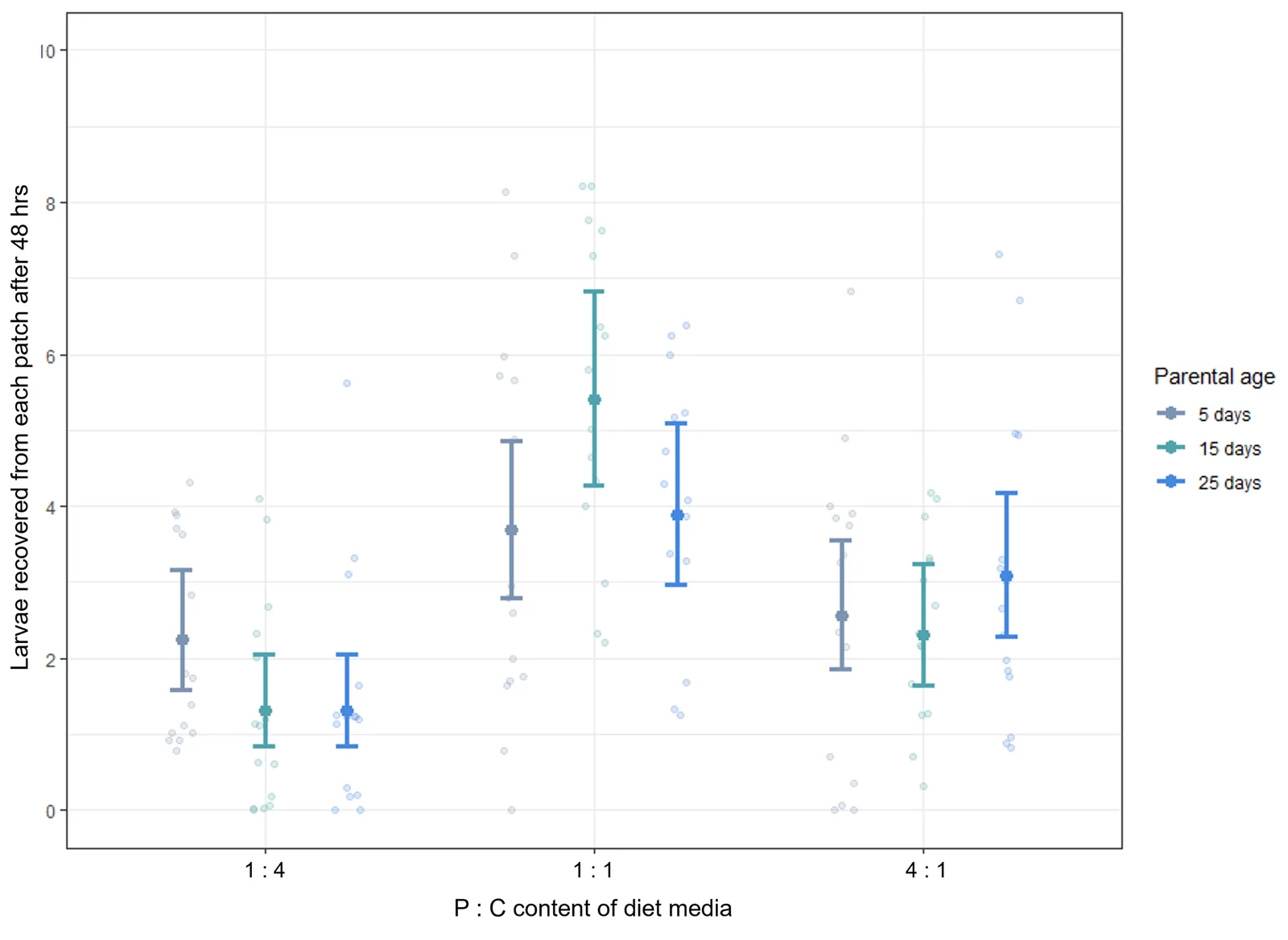
Number of larvae from 5, 15 and 25 d-old parents recovered from each diet patch after 48 h in choice assays (*N* = 150 per parental age group over 15 replicates per parental age group). A total of 450 larvae were transferred to choice assays within 24 h of hatching, of which 389 were recovered by manual extraction (equivalent to more than 8 out of the original 10 larvae per replicate, on average). Points show estimated marginal means and error bars represent 95% asymptotic (Wald-type) confidence intervals calculated in emmeans [55].

### (d) Parental age, nutrition and their interaction determined offspring survival to adulthood

Parental age had a strong main effect on the probability that offspring would eclose within 14 days of development, with offspring of 5 d parents possessing a significant survival advantage over offspring of 15 d parents (*χ^2^* = 64.9, d*f* = 1, *p* < 0.0001). Rearing diet also significantly predicted eclosion success in both parental age groups (*χ*^2^ = 15.8, d*f* = 2, *p* < 0.001). The interaction of parental age and rearing diet was also a significant determinant of offspring eclosion success (*χ*^2^ = 41.6, d*f* = 2, *p* < 0.0001; supplementary material, tables S11 and S12). Strikingly, offspring of 15 d parents began to eclose two full days later than offspring of 5 d parents on all diets (figure 4).

**Figure 4.**
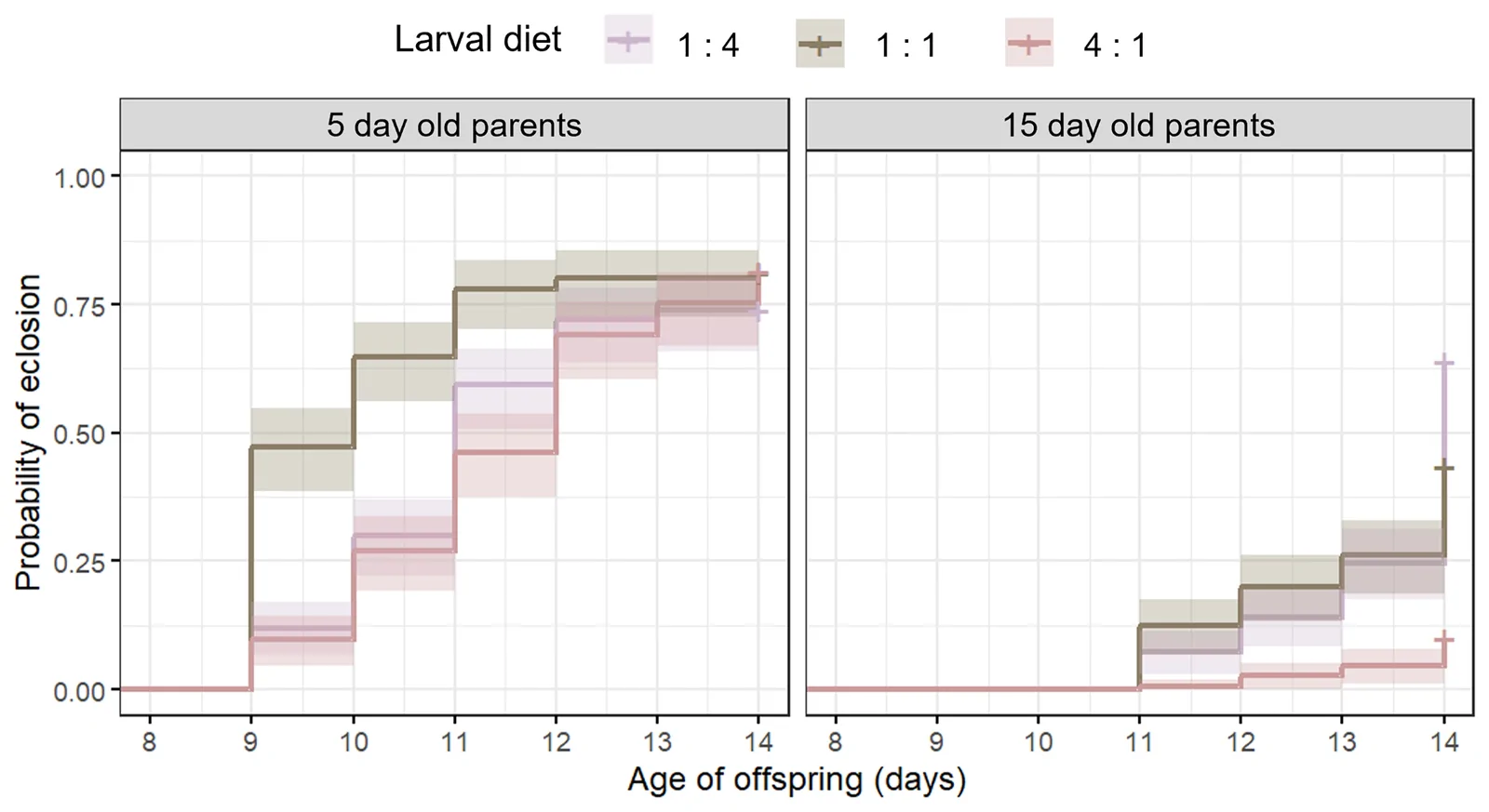
Probability that larvae reared on each of the three experimental diets under no-choice (*N* = 150 per parental age group per diet) would emerge from their pupae within 14 days when parents were 5 d (left) versus 15 d (right). Shown are Kaplan–Meier time-to-event curves based on time to eclosion in individual offspring that successfully eclosed (*N* = 523). As peak eclosion usually occurs at approximately 9 days, individuals that did not eclose by 14 days are likely to have died (*N* = 377).

Pairwise comparisons in *post-hoc* Tukey tests (supplementary material, tables S19 and S20) showed that eclosion probability was equal among diet groups in offspring with 5 d parents, despite the potential for protein toxicity on the 4 : 1 diet (*1 : 1 – 1 : 4* estimate = 0.50, *p* = 0.36; *1 : 1 – 4 : 1* estimate = 0.01, *p* = 1.00; *1 : 4 – 4 : 1* estimate = −0.49, *p* = 0.38). This was not the case in offspring with 15 d parents, in which survival was significantly higher on 1 : 4 than 1 : 1 (*1 : 1 – 1 : 4* estimate = −0.99, *p* = 0.01) or on 4 : 1 (*1 : 4 – 4 : 1* estimate = 3.20, *p* < 0.0001) and higher on 1 : 1 than on 4 : 1 (*1 : 1 – 4 : 1* estimate = 2.21, *p* < 0.0001).

Owing to low survivorship of individuals reared on 4 : 1 with 15 d parents, time to eclosion could only be analysed in the 1 : 4 and 1 : 1 diet groups. Results showed that larvae with 5 d parents reached eclosion faster than larvae with 15 d parents on both diet types (supplementary material, §S1.1, tables S10–S12, S21, and S22). Wing length, body size and shape showed expected sex differences and interactions between rearing diet and parental age (supplementary material, tables S13–S15 and S23–S25). The most consistent findings were for wing length, and to some extent body shape (wing length/thorax length), in which older (15 d) parents produced larger offspring of both sexes when offspring were reared on 1 : 4 and 4 : 1 diets, and smaller offspring on the 1 : 1 diet (supplementary material, figures S5 and S6). However, it is also possible that differences in preadult mortality may have contributed to the observed diet x parental age effects on morphology in adult offspring via selective disappearance (supplementary material, figures S4–S6, and §§S1.2 and S1.3).

Prediction no. 5 was that larval survival would be highest on the 1 : 1 P : C diet preferred by larvae and (sometimes) for egg-laying by females, and we did not predict a main effect of parental age. By contrast, our results showed that the probability of eclosion by 14 days in 5 d parent larvae was not affected by rearing diet, and in the 15 d parent group, probability of eclosion by 14 days was highest on the 1 : 4 diet, despite the developmental delay (supplementary material, §S1.1).

## 4. Discussion

In this study, we tested for sex-, age- and life stage-specific differences in diet preferences, and investigated how oviposition site choices of mothers of different ages influenced offspring fitness. The results showed significant effects of age and sex on diet patch selection, and that oviposition site choice in females was age-sensitive and only partially aligned with the diet choices of larvae. The preference of males for consistent high carbohydrate intake was as predicted. As expected, females showed a different pattern, and generally favoured diets containing higher levels of protein, although their preference for protein became stronger, not weaker as predicted, with age. Oviposition preferences were different from females' diet preferences, and did not fully overlap with the diet preferences of larvae. Specifically, young females preferred to lay eggs on a balanced (1 : 1) diet, with older females preferring higher carbohydrate. Interestingly, the direct tests of offspring development revealed that larval survival was not highest on the 1 : 1 P : C diet preferred by larvae (and sometimes but not always for egg-laying by females). We also found that a modest increase in parental age (from 5 to 15 d) led to significantly increased preadult mortality in offspring. In addition, preadult survival of offspring from 5 d parents was not significantly influenced by nutrition, whereas in offspring of 15 d parents, preadult survival had a strong relationship to dietary protein, with preadult survival maximized when protein was low. The results suggest that diet choices may not be completely optimized for parents and offspring, and reveal novel effects of parental age and offspring dietary environments in determining offspring survival.

### (a) Diet choice was sex- and age-specific, but did not follow predicted patterns

We predicted that females would favour high protein diets when young, and that this preference would diminish with age as their fertility declined. Males of all ages were expected to consistently favour high carbohydrate diets. Instead, although generally in line with predictions, diet preferences also varied with age in unexpected ways in both sexes. Males showed changing patterns of both patch occupancy (the choice to stand on any diet patch versus the dish) and diet patch choice with age. 25 d males favoured high protein and avoided the 1 : 1 balanced diet patch more than did 15 d or 5 d males, whereas preference for high carbohydrate diet patches remained consistent over age. One potential explanation is that males of all ages visited high carbohydrate patches to feed, while choosing to gather on other diet patches in the expectation that these sites were more likely to attract females. Also contrary to predictions, 5 d females did not prefer higher protein diets in comparison to 15 or 25 d females. Instead, the oldest (25 d) females visited the 4 : 1 diet more than both groups of younger females. It could be that multiple matings over a female's lifetime gradually increase protein appetite [17,20]. Counter to this idea, 15 d females visited the 1 : 4 diet more often than younger or older females. Overall, the results showed that the relationship between female age and dietary preference is nonlinear.

Measuring preference by patch occupancy (see §4e below) means that choices to be present on a specific diet patch versus on the dish might also be influenced by differences in social behaviour around resources. In all age groups, females were more likely to be observed on the dish rather than on a patch. By contrast, young and mid-age males (5 and 15 d) tended to congregate on patches for the duration of the assay. Male *D. melanogaster* are known to consume less food overall than females [17]. Therefore, rather than feeding, it is possible that 5 and 15 d males gathered on the food patches in anticipation of mating opportunities with females. Older (25 d) males did not show this pattern and instead more closely resembled females in being more frequently observed on the dish. This may reflect a reduction in competitive behaviour, perhaps arising from increased frailty or sperm depletion from multiple matings throughout the life course. Male diet choice was insensitive to mating rates in a study in which matings were controlled and occurred within a single day [20]. In the present study, males were co-housed with age-matched females under a 1 : 1 sex ratio throughout their lifetimes. The observed switch in patch choice behaviour could therefore have been influenced by the presence of same-sex competitors combined with age-related decline.

### (b) Oviposition preference for carbohydrate became stronger with age

Ovipositing females are known to continuously assess their surroundings and alter their egg-laying preferences according to the types of substrates available [58]. We hypothesized that oviposition site selection would become more variable as females aged, since selection acting to maintain advantageous egg-laying behaviours weakens after the onset of reproduction, as predicted by classical theories of ageing [59–62]. However, rather than showing weaker preference, older females laid a larger proportion of their eggs on the high carbohydrate 1 : 4 patch and fewer on the balanced 1 : 1 patch favoured by younger females.

Aversion to pure sucrose is well known in ovipositing females and has been exploited as an experimental paradigm by which to model simple decision-making [30,36,58,63]. Age-related decay in sucrose sensing pathways could provide a possible mechanism to explain older females' increased acceptance of, and attraction to, high sucrose media. Future work employing sucrose-based oviposition assays might benefit from accounting for female age as a confounding or explanatory variable.

The 5 d females preferred to lay eggs on the 1 : 1 diet patch but, unlike older cohorts, did not show any additional distinction between 1 : 4 and 4 : 1 patches. The higher fecundity of the youngest females might mitigate potential costs of placing eggs on suboptimal substrates and therefore allow for less selectivity. Alternatively, it may indicate that less preferred patches become increasingly acceptable as favoured sites become overloaded with other females' eggs, thus minimizing competition and density dependence, which may have been less intense in the less fecund 15 and 25 d cohorts. Future work could use video capture to record density-dependent changes in decision-making.

As larvae may be restricted in their ability to move to a more optimal patch after hatching, oviposition site choice predetermines the nutritional environment experienced by larvae for at least the initial stages of preadult development. In this way, changes in oviposition site choice by ageing females can be considered a form of behaviourally driven intergenerational maternal age effect. Such effects usually refer to epigenetic modifications, but non-genetic intergenerational effects can also encompass provisioned nutrients and transmitted behaviours [64,65]. Viewed through this lens, age-dependent changes in oviposition site preference could represent an underappreciated mechanism by which maternal age can shape offspring outcomes in insects, particularly if the choices of mothers and their offspring are not aligned.

### (c) Larvae chose a balanced diet, unless they had young parents

Larvae were expected to consistently favour the 1 : 1 diet patch (prediction no. 3), as balanced macronutrient intake is known to shorten larval development time [34,35] and larvae are attracted to intermediate, but repelled by high concentrations of sucrose [66]. This expectation was largely met, although larvae of young parents were more evenly distributed throughout the diet patches than those of older parents, potentially reflecting a greater tendency towards exploratory foraging behaviour in this group. Differences or imbalances in the condition of newly hatched older-parent larvae could also promote more specific intake trajectories as larvae attempt to compensate, explaining the more targeted feeding shown by older-parent larvae in choice assays.

### (d) Larval development followed expected patterns but also responded strongly to parental age

To evaluate the fitness effects of diet choices made by larvae and by ovipositing females, separate samples of larvae from 5 and 15 d parents were reared separately on each of the three test diets. Offspring were transferred onto experimental diets only after hatching. Therefore, differences in larval survival reflected intrinsic characteristics of the larvae rather than parent fertility. The 1 : 1 diet preferred by older-parent larvae, and by young ovipositing females, shortened larval development time but did not maximize larval survival, indicating a possible trade-off in which minimizing development time is more closely linked to higher fitness. Larvae of 5 d parents were equally likely to reach adulthood across all diets, whereas larvae of 15 d parents had the highest probability of survival on the 1 : 4 diet. The striking difference observed in survival seen between larvae of 5 versus 15 d parents was unexpected given the relatively modest difference in parental age. This result could indicate that even early reproductive ageing may carry consequences for offspring performance when exposed to suboptimal environments.

As parents were drawn from a freely mating, co-aged population, it is possible that constant mating opportunities caused a decline in gamete or ejaculate quality, affecting offspring phenotypes. In unmated males, periods of sexual rest, and presumably sperm build-up, can influence offspring phenotypes independently of paternal age, and seminal fluid protein/availability in older or multiply mated males is a stronger determinant of offspring number than sperm quantity *per se* [67,68]. Males could mate freely in the current study, so a build-up of sperm seems unlikely to have contributed to the observed decline in offspring quality. For mothers, variation in egg provisioning is a well-documented route by which maternal age can influence offspring outcomes, and could have mediated differences in offspring quality here, e.g. if 15 d mothers provisioned eggs with fewer or less balanced resources than 5 d mothers. Examples of such variation are known. For example, in the parasitoid wasp *Eupelmus vuilletti*, older mothers provision eggs with less protein and sugar [69,70].

Differences in provisioning quality could also explain the differences in morphology (wing and thorax length) observed across parent age and diet groups (supplementary material, §§S1.2 and S1.3). However, selective disappearance is also possible, given the high preadult mortality in some treatment groups. While the survival of older-parent offspring was reduced among all diet treatments here, shifts in parental provisioning may not always reduce viability or fitness. In the orb-weaving spider *Argiope radon*, older mothers produce fewer, heavier offspring that develop more slowly but exhibit greater starvation resistance in comparison with offspring of younger mothers [71]. It is possible that older parents, especially mothers, ‘recalibrate’ investment adaptively as their reproductive output declines, in a manner that confers advantage under some environmental conditions. In choice assays, older-parent larvae did not specifically avoid the 4 : 1 diet patch despite its apparent toxicity under no-choice conditions, whereas older mothers preferentially deposited eggs on the 1 : 4 substrate that yielded the highest survivorship among older-parent offspring in no-choice assays. This raises the possibility that older mothers may have been exercising ‘damage control’ by adaptively modifying their oviposition site selection to align with the most optimal patch for their differently provisioned offspring. Further work could usefully investigate the mechanisms underlying such parental age effects [44,72,73].

### (e) Diet preference testing

An important aspect of our study was to assess diet preferences by quantifying the presence of individuals on or in a diet patch, rather than the absolute amount of diet consumed. This means that we have indirect measures of diet preference (although patch presence is strongly associated with ingestion in females; [47]). Other methods for directly quantifying feeding preference and intensity are possible. For example, capillary-based methods such as the capillary feeder (CAFE) assay [74] and fly liquid-food interaction counter (FLIC) [75] can directly measure the intake of liquid foods in adults. However, these assays lack egg-laying substrates, making it impossible to compare feeding and oviposition preferences directly, which was a main aim of our study. They also cannot test larval diet preferences because larvae need a substrate through which to burrow. FlyPad [76] is another direct method for measuring proboscis extension and food intake via changes to electrical capacitance. However, the setup is optimized for testing two diets in adults and so was not suitable for evaluating preferences of larvae, or for oviposition choices. Dye uptake-type assays (e.g. [77]) can measure direct uptake of food from substrates by testing the rate and intensity with which a dye or label is detected in the abdomen of an adult fly. However, labels may differ in bioavailability [78] and sensitivity can be poor (low signal, making it difficult to detect subtle differences, especially for adult males and older flies that tend to eat less). In flies that are feeding, a saturated steady state is quickly reached, introducing a time lag between what was, and is, being eaten. However, these methods—and even direct observations [16]—could now be used to build upon the results shown here to refine measures of adult preferences with age.

Testing of flies of different ages was done on flies maintained up until each respective age on standard SYA medium. This assayed the preferences of naive flies that had not previously been exposed to the test diets—at young, mid and older ages—to detect evidence for ageing in diet preferences. Future work could examine whether there is any interaction between the length of exposure to the standard SYA diet and the subsequent results of the preference testing on different diets. The types of experimental diets used here are also unlikely to represent the dietary environment of the flies in natural settings. However, fruits are high in sugar and become progressively more protein-rich as yeasts colonize them [79], so varying from carbohydrate-rich to protein-rich is relevant, even though the absolute parameters are likely to be different. However, it cannot be ruled out that preferences might be maladaptive as a result of constraints on the capacity for evolution to equip flies for this specific type of diet choice. Flies in nature may rarely have the extent of choice available here, and this would repay further study.

## Supplementary Material



## Data Availability

Raw data and code are available via a Zenodo repository [80]. Supplementary material is available online [81].
