# Supplementary material for: Age-related shifts in dietary and oviposition preferences and steep costs of modest parental ageing in Drosophila melanogaster
Source: Proc Biol Sci. 2026 Nov 11;293(2082):20261983. doi: 10.1098/rspb.2026.1983 (PMC13751124; doi:10.1098/rspb.2026.1983)
Supplement: Supplementary file 1 [file rspb.2026.1983.zip › rspb.2026.1983/rspb20261983_si_001.pdf]

Supplementary information: Age-related shifts in dietary and oviposition preferences and steep costs of modest parental ageing in Drosophila melanogaster

Oonagh Barker[1], Edward R. Ivimey-Cook[1], Wilfried Haerty[2], Alexei A. Maklakov[1] and Tracey Chapman[1]

1 School of Biological Sciences, University of East Anglia, Norwich, Norfolk, UK, NR47TJ

2 Earlham Institute, Norwich Research Park, Norwich, Norfolk, UK, NR4 7UZ

Journal: Proceedings of the Royal Society B

Paper DOI: 10.1098/rspb.2016.0049

Data and code repository DOI: 10.5281/zenodo.22235767

1. Supplementary results

1.1 Parental age and diet determined larval development time under no-choice rearing conditions (1:4 and 1:1 diets only)

Parental age had a significant main effect on time to eclosion in both the 1:4 and 1:1 diets (X^2 = 321.5, df = 1, p <0.0001). Rearing diet also had a significant effect on development time (X^2 = 22.6, df = 1, p < 0.0001). The interaction of parental age and rearing diet was marginally nonsignificant (X^2 = 3.51, df = 1, p = 0.06; Table S11).

Pairwise comparisons (Tables S21-S22) showed that larvae of 5d parents reached eclosion faster than larvae of 15d parents on both diets (1:4 reared larvae: *15d parents – 5d parents* estimate = 0.22, p <0.0001; 1:1 reared larvae: *15d parents – 5d parents* estimate = 0.27, p <0.0001). Larvae of 5d parents showed faster development on 1:1 than on 1:4 , as expected in prediction #5 (*1:1 – 1:4* estimate = -0.09, p <0.0001). However, this effect was marginally nonsignificant in larvae of 15d parents (*1:1 – 1:4* estimate = -0.04, p = 0.06).

1.2 Parental age interacts with nutrition to determine offspring wing and thorax size

Wing length (Figure S3a) was determined by a main effect of sex, as expected (X^2 = 509, df = 1, p <0.0001), a main effect of rearing diet (X^2 = 22.1, df = 2, p <0.0001) and an interaction between rearing diet and parental age (X^2 = 103, df = 2, p <0.0001; Table S14).

Pairwise comparisons (Table S23) showed that wing size in males and females responded similarly to diet and parental age, remaining proportionate to the size difference between the sexes. In individuals with 15d parents, wing size was smaller than in individuals with 5d parents when reared on 1:1 (Males: estimate = -0.17, p <0.0001; Females: estimate -0.23, p <0.0001), but larger than in individuals with 5d parents when reared on 1:4 (Males: estimate 0.12, p <0.001; Females: estimate 0.14, p <0.0001). A sex difference emerged in wing length responses

to 4:1 by parental age in that sons of 15d parents had significantly longer wings than sons of 5d parents (estimate = 0.13, p = 0.001) while this effect was marginally nonsignificant in daughters (estimate = 0.08, p = 0.08).

Thorax length (Figure S3b) showed the expected strong effect for sex ($X^2 = 496$, df = 1, p <0.0001) and a main effect of rearing diet ($X^2 = 14.5$, df = 2, p <0.001), and an interaction between rearing diet and parental age ($X^2 = 7.55$, df = 2, p = 0.02; Table S13).

Pairwise comparisons (Table S24) revealed that patterns of thorax size did not completely match wing length leading to differences in proportionality by treatment group (SI 1.3). Females reared on 1:1 with 15d parents had smaller thoraxes than females reared on 1:1 with 5d parents (estimate = -0.04, p = 0.04) while there was no parental age difference in thorax size in individuals reared on 1:4 (estimate = 0.02, p = 0.24) or 4:1 (estimate = -0.03, p = 0.33). Meanwhile, males with 15d parents reared on 1:4 had larger thoraxes than males with 5d parents reared on 1:4 (estimate = 0.06, p <0.01). This was marginally nonsignificant in males reared on 4:1 (estimate = 0.05, p = 0.06) and absent in males reared on 1:1 (estimate = -0.002, p = 0.92).

1.3 Parental age and rearing diet exert sex-specific effects on offspring body shape

Like the analyses of wing and thorax length, there was a significant effect for the interaction of parental age and rearing diet ($X^2 = 10.2$, df = 2, p <0.01). A marginal effect was also detected for an interaction of sex by parental age in determining wing/ thorax proportionality ($X^2 = 3.77$, df = 1, p = 0.051; Table S15).

Sexual dimorphism was also detected in the ratio of wing length to thorax length; males had longer wings relative to their thoraxes, and females had shorter wings relative to their thoraxes ($X^2 = 47.7$, df = 1, p < 0.0001).

Pairwise comparisons (Table S25) in post-hoc Tukey tests showed more varied responses in females than in males (Figure S4). Both male and female offspring of 15d parents reared on 1:1 had smaller wings/ larger thoraxes than offspring of 5d parents (Males: estimate -0.19, p = 0.03; Females: estimate -0.15, p = 0.02), while female offspring of 15d parents reared on 4:1 had larger wings/ smaller thoraxes than female offspring of 5d parents reared on 4:1 (estimate = 0.18, p = 0.03).

2. Supplementary methods

2.1 Body size measures

Frozen offspring vials were removed from the freezer in batches and every individual (N = 523) was photographed using an Eagle-M microscope with a GXCAm screen. Photographs were taken in lateral view which provides clear visibility of the thorax and wings (Figure S2). All measurements were taken in imageJ v1.54i. To analyse the effects of rearing diet and parental age on body proportions, wing length was divided by thorax length for each individual to obtain a measurement of proportionality. Individuals with damaged or awkwardly positioned wings were included in thorax analyses but excluded from wing length and proportionality analyses (N = 8).

3. Supplementary figures

Figure S1

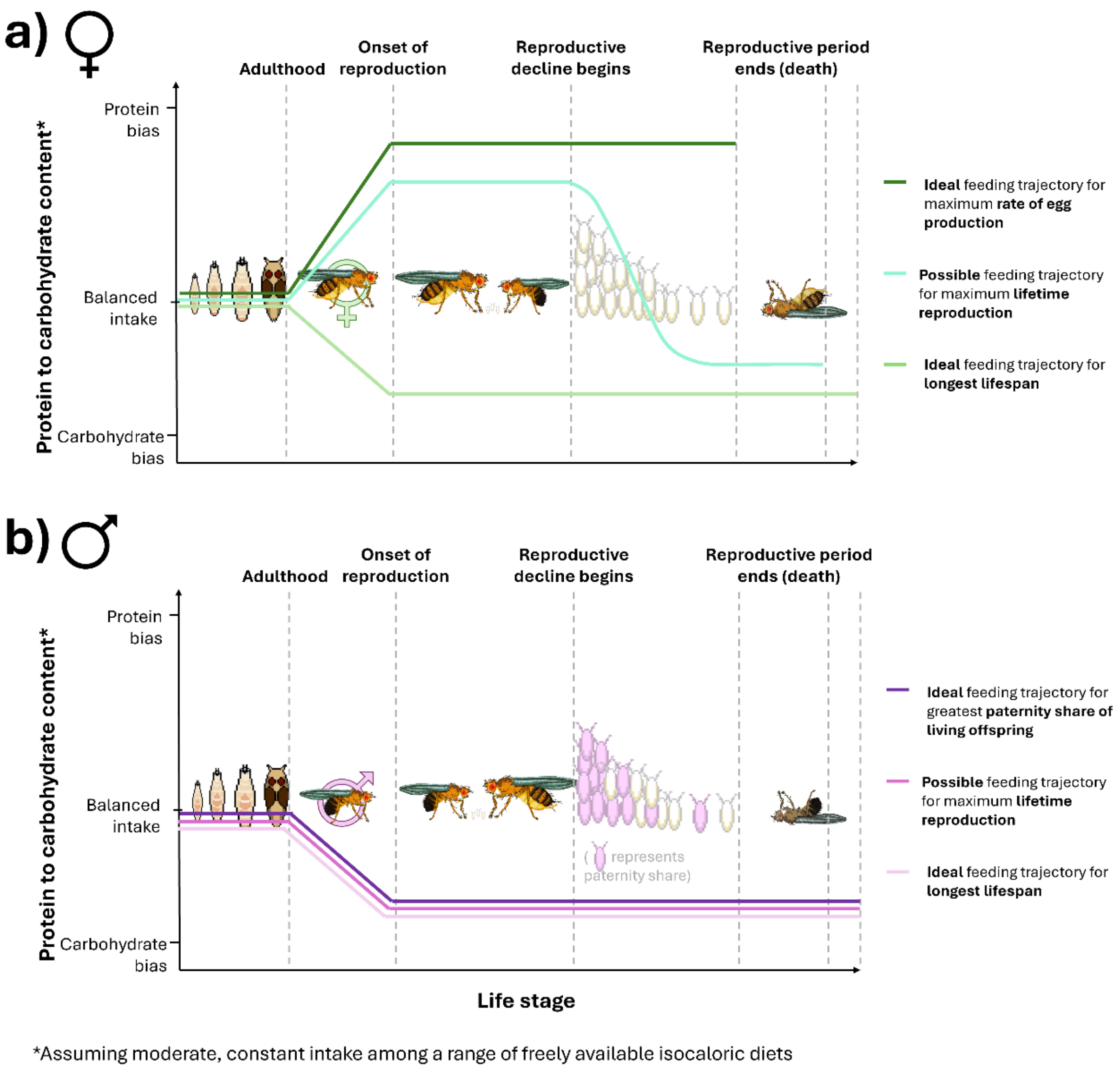

Figure S1: A schematic visualisation comparing how each sex might adjust feeding trajectory over the life course. For simplicity, this assumes constant availability of a range of isocaloric food sources over a gradient of high protein to high carbohydrate content. **a**: Hypothesised intake trajectories which could be taken by females. The **dark green/top** trajectory would maximise the rate of egg production and result in shorter lifespan; the **blue-green/middle** trajectory could support both high egg production in early life and extend reproductive lifespan, potentially leading to maximised lifetime reproduction; lastly, the male-like **pale green/bottom** trajectory would maximise lifespan at cost to reproduction. **b**: Hypothesised intake trajectories which could be taken by males. Unlike females, the same high-carbohydrate and low-protein trajectory is predicted to benefit short-term reproductive rate, understood here as paternity share of eggs at any given time (**purple/top**), lifetime reproductive output (**hot pink/middle)**, and lifespan (**light pink/bottom)**.

Figure S2

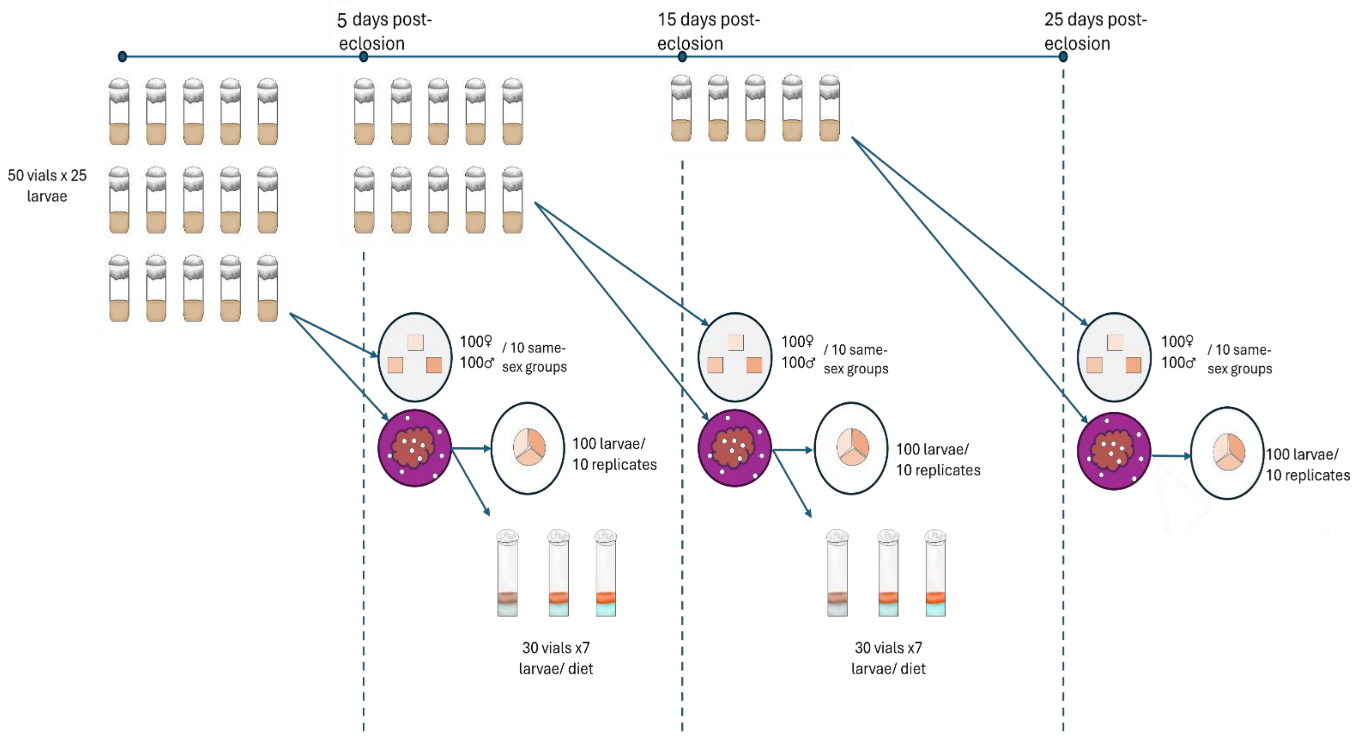

Figure S2: **Schematic illustrating how individuals were sampled at three time-points from a large, co-aged population for assays of adult dietary choice, larval dietary choice and larval development under no-choice.** We first established a large, low density cohort of standardised parental age by transferring larvae of 5 day old parents to 50 vials of SYA in groups of 25 (N = 1,250). After eclosion, individuals were transferred to fresh SYA vials in groups of 10 males and 10 females (N = 1,200), and thereafter were transferred to fresh media twice weekly until the end of the experiment. At 5d post eclosion, one third of this cohort (N = 400 individuals in 20 vials) were selected at random and split into two groups. One group (N = 200) was used for adult diet choice assays, while eggs were collected from the other group (N = 200) over a 24hr period on plates of purple grape juice agar. Eggs from the latter group were incubated for a further 24hrs, and then first instar larvae were collected for larval choice assays and assays of larval development under no-choice on the 1:4, 1:1 and 4:1 experimental diets. At 15d post eclosion, a further 400 individuals were randomly sampled from the large starting cohort, split into two groups of 200 individuals, and these groups were used again for either adult choice assays or egg collection for larval diet choice and larval non-choice development assays. At 25d post eclosion, the remaining individuals (approximately 380) were again assigned at random to the diet choice group

Figure S3

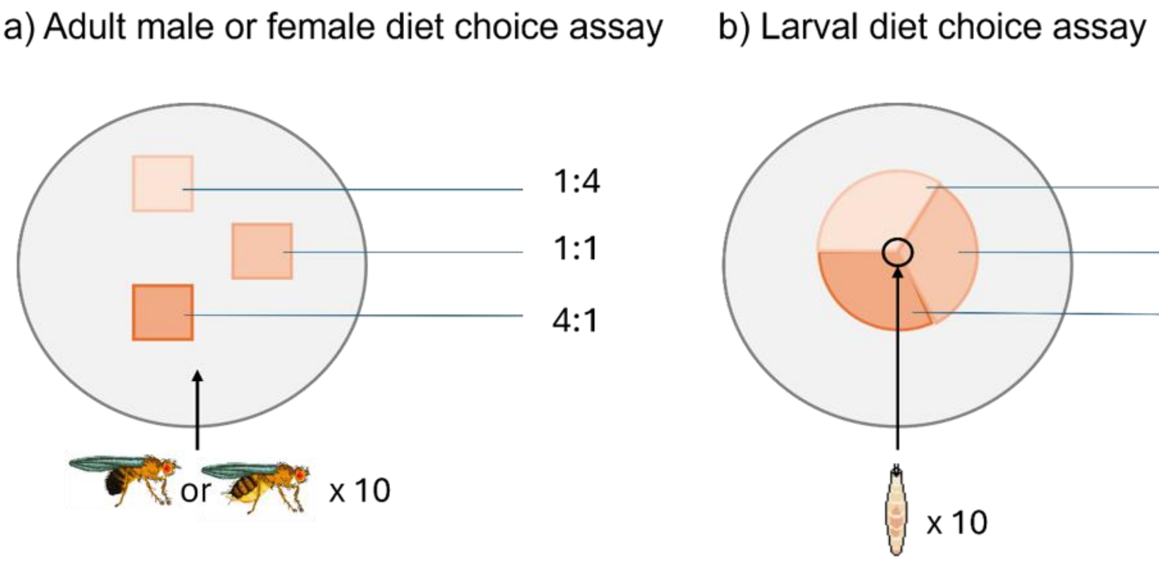

Figure S3: **Illustration showing how three-choice assays were configured for adult males or females (a) and for larvae (b).** These utilised synthetic solid diets with macronutrient concentrations at 1:4, 1:1 and 4:1 parts protein (casein) to carbohydrate (sucrose). **A**: Choice assay setup used for single-sex groups of 10 adult males or females at 5, 15 and 25d after eclosion. Flies were introduced to the plates under light CO_2 sedation and allowed 1h to recover and acclimatise, after which we recorded the number of flies gathered on each diet patch or on no patch (any individuals standing on the sides or edges of the plate, not touching any diet patch) at half-hourly intervals over a period of 4h. **B**: Choice assay setup used for larvae. Eggs were collected from the parental cohort at 5, 15, and 25d post eclosion on plates of purple grape juice agar. After hatching, groups of 10 first instar larvae were collected from the purple grape plates and placed at the centre of the choice assay plates where the three diet patches intersect. After a period of 48hrs of free foraging, the diet patches were pulled apart and the larvae within each patch were manually extracted and counted to assess diet choice.

Figure S4

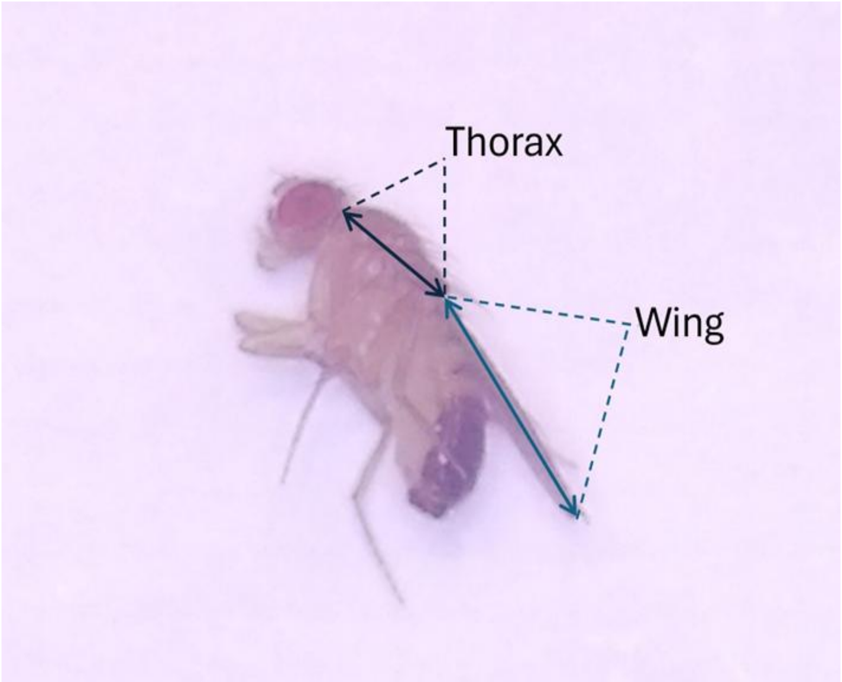

Figure S4: **An example photograph from the dataset for wing and thorax measures of the eclosed offspring from the non-choice development experiment, with markers illustrating the measurement points.** Thorax length (in mm) was measured from the back of the head to the ’ridge’ at the end of the thorax which is well-defined in lateral view. Wing measurements were taken from this point to the end of the closest wing, and only when wings were undamaged and held in the folded position pictured.

Figure S5

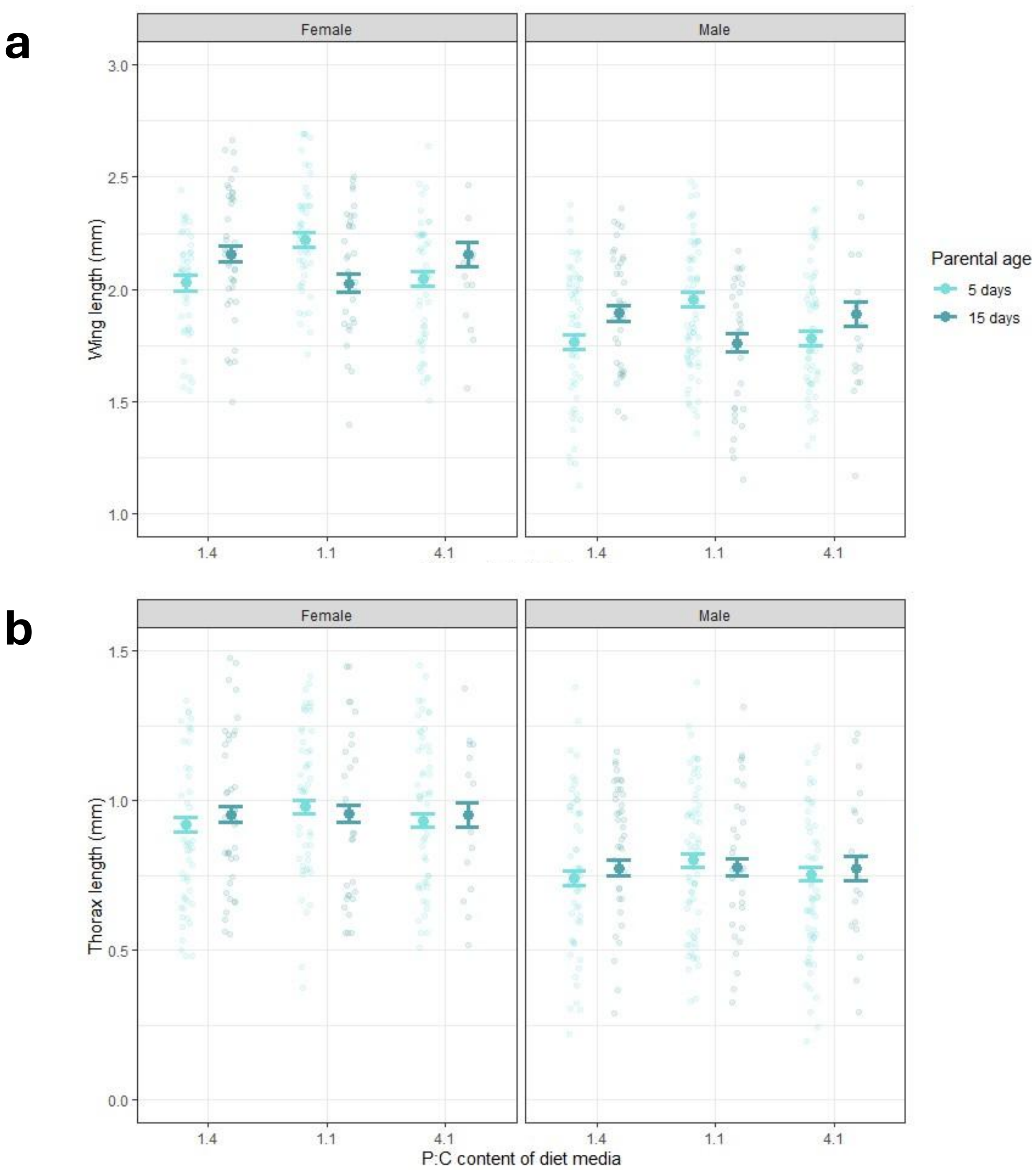

Figure S5: A: wing length and B: thorax length in male and female offspring of 5d parents (N = 341) versus 15d parents (N = 182) when reared under no-choice on the three experimental diets. a: Shown is wing length in mm measured from the base to the tip of the wing in lateral view for each individual (Figure S2). Offspring of 5d parents had longer wings than offspring of 15d parents if offspring were raised on the 1:1 P:C diet, but smaller wings than 15d parents if offspring were raised on 1:4 or 4:1 P:C diets. This pattern was present in both sexes. **b:** Shown is thorax length in mm measured from the back of the head to the base of the wing in lateral view (Figure S2). In females, there was no difference in thorax size by parental age. There was no difference in thorax size by parental age in male offspring reared on the 1:1 P:C diet, but male offspring of 5d parents reared on the 1:4 or 4:1 P:C diets had smaller thoraxes than male offspring of 15d parents. Taken together, these wing and thorax size differences led to differences in proportionality among parental age and offspring rearing diet groups (SI 3.3). Points show estimated marginal means and error bars represent 95% asymptotic (Wald-type) confidence intervals calculated in emmeans (Lenth, 2023).

Figure S6

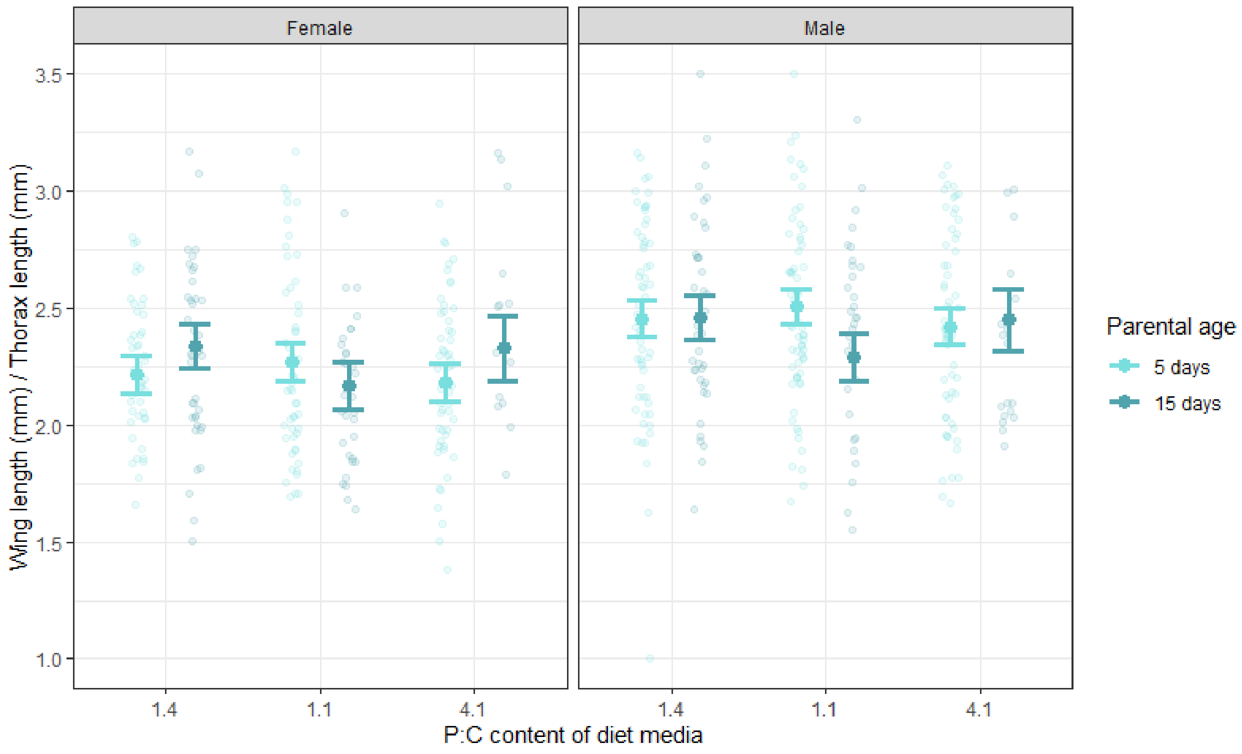

Figure S6: Wing length (mm) divided by thorax length (mm) in offspring of 5d (N = 341) versus 15d parents (N = 182) reared on the three experimental diets. Larger values indicate longer wings relative to thoraxes. In both males and females, offspring of 15d parents reared on 1:1 has smaller wings and larger thoraxes relative to offspring of 5d parents reared on 1:1. In addition, female offspring of 15d parents reared on 4:1 had larger wings and smaller thoraxes than female offspring of 5d parents reared on 4:1. Points show estimated marginal means and error bars represent 95% asymptotic (Wald-type) confidence intervals calculated in emmeans (Lenth, 2023).

4. Supplementary tables

4.1 Meridic diet recipes

Table S1: Recipe for 1L each of the sucrose-casein meridic diets

**Ingredient**	**1:4**	**1:1**	**4:1**
Casein(g)	24	60	96
Sucrose (g)	96	60	24
Agar(g)	100	100	100
Cholesterol(g)	100	100	100
Lecithin(g)	100	100	100
K2HPO4(ml)	100	100	100
KH2PO4(ml)	100	100	100
MgSO4(ml)	100	100	100
MaHCO3(ml)	100	100	100
Nucleic acid(ml)	100	100	100
Deioinised water(ml)	200	200	200
Vitamin mix(ml)	150	150	150
Nipagin(ml)	10.0	10.0	10.0
Propionic acid(ml)	3.00	3.00	3.00

Table S2: Recipe for 1L nucleic acid solution used in experimental diets

Ingredient	Quantity
Uridine(g)	5.70
Inosine(g)	6.40
Deinonised water	1000

Table S3: Recipe for 1L vitamin mix solution used in experimental diets

Ingredient	Quantity
Thiamine(g)	0.013
Riboflavin(g)	0.066
Nicotinic acid(g)	0.800
Calcium pantothenate(g)	0.111
Pyridoxine(g)	0.016
Biotin solution(ml)	66.50
Folic acid solution(ml)	66.50
Deionised water(ml)	866.5

Table S4: Recipe for 1L biotin solution used in vitamin mix

Ingredient	Quantity
Biotin(g)	0.02
Deionised water(ml)	1000

Table S5: Recipe for 1L folic acid solution used in vitamin mix

Ingredient	Quantity
Folic acid(g)	0.075
Ethanol(ml)	200
Deionised water(ml)	800

4.2 Model table

Table S6: Model families by level of individual and traits of interest

**Model family**	**Level of individual**	**Trait of interest**	**Fixed effects**	**Random effects**
Negative binomial	P0 (mated males and females)	Diet choice	Sex, age, diet patch	Replicate (plate of 10 individuals), observation time
Beta-binomial	P0 (mated females)	Oviposition site choice	Age, diet patch	Replicate (plate of 10 individuals), observation (per diet patch per plate)
Poisson	F1 (larvae)	Diet choice	Diet patch, parental age	Replicate (plate of 10 individuals), observation (per diet patch per plate)
Cox Proportional Hazards	F1 (larvae)	Egg-to-adult development time	Diet patch, parental age	Vial (of 5 individuals), individual ID
Conway-Maxwell Poisson	F1 (larvae)	Egg-to-adult development time	Diet patch, parental age	Vial (of 5 individuals), individual ID
Binomial	F1 (larvae)	Preadult mortality	Diet patch, parental age	Vial (of 5 individuals)
Gaussian	F1 (adults)	Thorax length	Sex, diet patch, parental age	Vial (of 5 individuals), individual ID
Gaussian	F1 (adults)	Wing length	Sex, diet patch, parental age	Vial (of 5 individuals), individual ID
Gaussian	F1 (adults)	Wing length/ thorax length (proportionality)	Sex, diet patch, parental age	Vial (of 5 individuals), individual ID

4.3 Full model outputs

Table S7

Table S7: Full model output for diet choice over the age course in mated males and females at 5, 15 and 25d. Shown here is the summary of the negative binomial generalised linear mixed effects model for all fixed effects. Random effects were included for replicate and observation time, and a zeroinflation parameter was used to model zeroinflation as a constant probability across all observations (“~1”).

**Predictor**	**Estimate**	**Std. error**	**Z value**	**Pr (>|z|)**
(Intercept)	0.11935	0.09672	1.234	0.217213
Sex (Male)	0.81023	0.12148	6.670	2.56e-11 ***
Age (25d)	-0.25932	0.14646	-1.771	0.076626 .
Age (5d)	0.43498	0.12382	3.513	0.000443 ***
Patch (1:4)	-0.22882	0.14465	-1.582	0.113657
Patch (no patch)	1.75844	0.10459	16.812	<2e-16 ***
Patch (4:1)	0.37042	0.12548	2.952	0.003158 **
Sex (Male) * Age (25d)	-1.06486	0.20654	-5.156	2.53e-07 ***
Sex (Male) * Age (5d)	-0.78297	0.16300	-4.804	1.56e-06 ***
Sex (Male) * Patch (1:4)	-0.16957	0.18039	-0.940	0.347223
Sex (Male) * Patch (no patch)	-1.51734	0.13878	-10.933	<2e-16 ***
Sex (Male) * Patch (4:1)	-0.29690	0.15793	-1.880	0.060115 .
Age (25d) * Patch (1:4)	-0.32888	0.23149	-1.421	0.155407
Age (5d) * Patch (1:4)	-0.79283	0.20862	-3.800	0.000145 ***
Age (25d) * Patch (no patch)	0.34859	0.15636	2.229	0.025790 *
Age (5d) * Patch (no patch)	-0.40964	0.13574	-3.018	0.002546 **
Age (25d) * Patch (4:1)	0.27675	0.18514	1.495	0.134970
Age (5d) * Patch (4:1)	-0.79989	0.17576	-4.551	5.34e-06 ***
Sex (Male) * Age (25d) * Patch (1:4)	1.39331	0.29986	4.647	3.38e-06 ***
Sex (Male) * Age (5d) * Patch (1:4)	1.11748	0.26220	4.262	2.03e-05 ***
Sex (Male) * Age (25d) * Patch (no patch)	1.63735	0.22446	7.295	3.00e-13 ***
Sex (Male) * Age (5d) * Patch (no patch)	0.95372	0.18926	5.039	4.68e-07 ***
Sex (Male) * Age (25d) * Patch (4:1)	0.64145	0.25708	2.495	0.012592 *
Sex (Male) * Age (5d) * Patch (4:1)	1.12765	0.22613	4.987	6.14e-07 ***

Table S8

Table S8: Full model output for oviposition site selection by mated females at 5, 15 and 25d. Eggs laid on each patch (“successes”) Shown here is the summary of the betabinomial generalised linear mixed effects model for all fixed effects. To control for overdispersion and zeroinflation caused by declining fertility with age, a two-column response matrix was constructed by treating eggs paid on each patch in a replicate as “successes” and eggs laid on all other patches as “failures”. This response matrix was used as the response variable. Random effects were included for observation (an observation-level random effect for each patch) and replicate.

**Predictor**	**Estimate**	**Std. error**	**Z value**	**Pr(>|z|)**
(Intercept)	1.71328	0.19507	8.782	1.59e-18 ***
Age (25d)	-1.41810	0.25998	-5.454	4.91e-08 ***
Age (5d)	-0.07728	0.26402	-0.292	0.769744
Patch (1:4)	-3.87531	0.30165	-12.846	8.95e-38 ***
Patch (4.1)	-4.92688	0.36387	-13.539	9.08e-42 ***
Age (25d) * Patch (1.4)	3.01899	0.38018	7.940	2.01e-15 ***
Age (5d) * Patch (1.4)	-0.08327	0.38679	-0.215	0.829548
Age (25d) * Patch (4.1)	1.73508	0.43052	4.030	5.57e-05 ***
Age (5d) * Patch (4.1)	0.63836	0.42407	1.505	0.132251

Table S9

Table S9: Full model output for diet choice in larvae of 5, 15 and 25d parents (both parent sexes age-matched). Shown here is the summary of the Poisson generalised linear mixed effects model for all fixed effects. Random effects were included for replicate and observation (an observation-level random effect for each patch).

**Predictor**	**Estimate**	**Std. error**	**Z value**	**Pr(>|z|)**
(Intercept)	1.6856	0.1206	13.980	<2e-16 ***
Patch (1:4)	-1.4117	0.2567	-5.499	3.83e-08 ***
Patch (4:1)	-0.8516	0.2109	-4.038	5.39e-05 ***
Parental age (25d)	-0.3123	0.1805	-1.730	0.08356 .
Parental age (5d)	-0.3823	0.1838	-2.080	0.03748 *
Patch (1:4) * Parental age (25d)	0.3606	0.3659	0.985	0.32441
Patch (4:1) * Parental age (25d)	0.6277	0.2927	2.144	0.03201 *
Patch (1:4) * Parental age (5d)	0.9133	0.3419	2.671	0.00756 **
Patch (4:1) * Parental age (5d)	0.4897	0.3028	1.617	0.10581

Table S10

Table S10: Full model output from Cox proportional hazards model for egg-to-adult development time in larvae of 5 and 15d parents reared on 1:4, 1:1 or 4:1 P:C diets. Shown here is the summary of fixed effects from the Cox proportional hazards model, which included fixed effects for parental age and diet and random effects for vial (5 individuals per vial) and individual ID.

**Predictor**	**coef**	**exp(coef)**	**se(coef)**	**z**	**p**
Parental age	-0.27776	0.75748	0.02878	-9.65	<2e-16
Diet (1:4)	-1.81920	0.16215	0.43086	-4.22	2.42e-05
Diet (4:1)	-0.68032	0.50645	0.45005	-1.51	0.13062
Parental age * Diet (1:4)	0.14908	1.16076	0.03949	3.77	0.00016
Parental age * Diet (4:1)	-0.07998	0.92314	0.04695	-1.70	0.08846

Table S11

Table S11: Full model output from the Conway-Maxwell Poisson generalised linear mixed effects model for egg-to-adult development time in larvae of 5 and 15d parents reared on 1:4 or 1:1 P:C diets; the 4:1 diet had to be excluded from this analysis due to convergence problems which arose from high preadult mortality in larvae of 15d parents reared on this diet. Shown here is the summary of fixed effects from the Conway-Maxwell Poisson model, which included fixed effects for parental age and diet and random effects for vial and individual ID.

**Predictor**	**Estimate**	**Std. error**	**Z value**	**Pr(>|z|)**
(Intercept)	2.54844	0.01555	163.89	<2e-16 ***
Parental age (5d)	-0.27460	0.02062	-13.31	<2e-16 ***
Diet (1:4)	0.03921	0.02051	1.91	0.0559 .
Parental age (5d) * Diet (1:4)	0.05170	0.02758	1.87	0.0608 .

Table S12

Table S12: Full model output from the binomial generalised linear mixed effects model for preadult mortality (event history analysis) in larvae of 5 and 15d parents reared on 1:4, 1:1 or 4:1 P:C diets. Shown here is the summary of fixed effects. A random effect was included for vial.

Predictor	Estimate	Std. error	Z value	Pr(>|z|)
(Intercept)	1.677082	0.278507	6.022	1.73e-09 ***
Parental age (15d)	-1.983303	0.368951	-5.376	7.64e-08 ***
Diet (1:4)	-0.502359	0.369950	-1.358	0.174492
Diet (4:1)	-0.007976	0.386525	-0.021	0.983538
Parental age (15d) * Diet (1:4)	1.487628	0.502203	2.962	0.003054 **
Parental age (15d) * Diet (4:1)	-2.204381	0.569394	-3.871	0.000108 ***

Table S13

Table S13: Full model output from the gaussian generalised linear mixed effects model for thorax length in adult offspring of 5 and 15d old parents. Offspring were reared on 1:4, 1:1 and 4:1 P:C diets. Shown here is the summary of fixed effects. Random effects were included for vial and an observation-level random effect was included for each individual.

Predictor	Estimate	Std. error	Z value	Pr(>|z|)
(Intercept)	0.958997	0.013992	68.54	<2e-16 ***
Diet (1:4)	-0.003934	0.018060	-0.22	0.82756
Diet (4:1)	-0.007756	0.023110	-0.34	0.73717
Parental age (5d)	0.022072	0.016890	1.31	0.19128
Sex (Male)	-0.182312	0.008187	-22.27	<2e-16 ***
Diet (1:4) * Parental age (5d)	-0.064289	0.023427	-2.74	0.00607 **
Diet (4:1) * Parental age (5d)	-0.038506	0.027403	-1.41	0.15997

Table S14

Table S14: Full model output from the gaussian generalised linear mixed effects model for wing length in adult offspring of 5 and 15d old parents. Offspring were reared on 1:4, 1:1 and 4:1 P:C diets. Shown here is the summary of fixed effects. Random effects were included for vial and an observation-level random effect was included for each individual. Nonsignificant interactions were retained in this case as they slightly improved model fit (AIC).

Predictor	Estimate	Std. error	Z value	Pr(>|z|)
(Intercept)	2.021351	0.026405	76.55	<2e-16 ***
Diet (1:4)	0.132780	0.035177	3.77	0.00016 ***
Diet (4:1)	0.110637	0.046099	2.40	0.01640 *
Parental age (5d)	0.228220	0.033422	6.83	8.58e-12 ***
Sex (Male)	-0.252898	0.034855	-7.26	4.00e-13 ***
Diet (1:4) * Parental age (5d)	-0.370404	0.045887	-8.07	6.91e-16 ***
Diet (4:1) * Parental age (5d)	-0.315623	0.054233	-5.82	5.89e-09 ***
Diet (1:4) * Sex (Male)	-0.005141	0.045767	-0.11	0.91056
Diet (4:1) * Sex (Male)	0.030846	0.058461	0.53	0.59776
Parental age (5d) * Sex (Male)	-0.061697	0.042454	-1.45	0.14616
Diet (1:4) * Parental age (5d) * Sex (Male)	0.086952	0.057944	1.50	0.13345
Diet (4:1) * Parental age (5d) * Sex (Male)	0.022434	0.067919	0.33	0.74117

Table S15

Table S15: Full model output from the gaussian generalised linear mixed effects model for wing length divided by thorax width in adult offspring of 5 and 15d parents. Offspring were reared on 1:4, 1:1 and 4:1 P:C diets. Shown here is the summary of fixed effects. Random effects were included for vial and an observation-level random effect was included for each individual.

**Predictor**	**Estimate**	**Std. error**	**Z value**	**Pr(>|z|)**
(Intercept)	2.16526	0.05220	41.48	< 2e-16 ***
Sex (Male)	0.12065	0.04958	2.43	0.01495 *
Parental age (5d)	0.10215	0.06604	1.55	0.12192
Diet (1:4)	0.16937	0.06171	2.74	0.00605 **
Diet (4:1)	0.16250	0.07943	2.05	0.04076 *
Sex (Male) * Parental age (5d)	0.11884	0.06116	1.94	0.05200 .
Parental age (5d) * Diet (1:4)	-0.22297	0.07970	-2.80	0.00515 **
Parental age (5d) * Diet (4:1)	-0.24907	0.09356	-2.66	0.00777 **

4.4 Full outputs of emmeans Tukey tests

Table S16

Table S16: Post-hoc Tukey tests comparing how different age groups of mated males and females interacted with diet choice assays.

	Contrast	Estimate	SE	Z ratio	p value
In males					
Landing on 1:4					
	25d - 5d	-0.21	0.14	-1.51	0.29
	15d – 5d	0.04	0.13	0.31	0.29
	15 d - 25d	0.25	0.14	1.83	0.16
Landing on 1:1					
	25d - 5d	-0.95	0.16	-5.91	<0.0001
	15d - 5d	0.34	0.12	2.86	0.01
	15d - 25d	1.29	0.15	8.17	<0.0001
Landing on 4:1					
	25d - 5d	-0.38	0.12	-3.26	<0.01
	15d - 5d	0.02	0.10	0.18	0.98
	15d - 25d	0.40	0.11	3.46	<0.01
No patch					
	25d - 5d	0.46	0.07	6.30	<0.0001
	15d - 5d	-0.18	0.09	-1.99	0.11
	15d - 25d	-0.64	0.08	-7.63	<0.0001
In females					
Landing on 1:4					
	25d - 5d	-0.25	0.18	-1.40	0.34
	15d - 5d	0.35	0.15	2.32	0.05
	15d - 25d	0.60	0.16	3.65	<0.001
Landing on 1:1					
	25d - 5d	-0.67	0.12	-5.61	<0.0001
	15d - 5d	-0.44	0.11	-3.97	<0.001
	15d - 25d	0.23	0.13	1.76	0.18
Landing on 4:1					
	25d - 5d	0.40	0.11	3.49	0.001
	15d - 5d	0.39	0.11	3.39	0.002
	15d - 25d	-0.01	0.10	-0.10	0.99
No patch					

25d - 5d	0.06	0.04	1.3	0.39
15d - 5d	-0.02	0.05	-0.48	0.88
15d - 25d	-0.089	0.05	-1.80	0.17

Table S17

Table S17: Post-hoc Tukey tests comparing the proportion of eggs laid on each diet choice patch by mated females at 5, 15 and 25d post-eclosion.

	Contrast	Estimate	SE	df	Z ratio	p value
Eggs laid on 1:4						
	15d - 25d	-1.60	0.27	Inf	-5.97	<0.0001
	15d - 5d	0.16	0.28	Inf	0.58	0.82
	25d - 5d	1.76	0.26	Inf	6.27	<0.0001
Eggs laid on 1:1						
	15d - 25d	1.41	0.26	Inf	5.45	<0.0001
	15d - 5d	0.08	2.72	Inf	0.29	0.95
	25d - 5d	-1.33	0.26	Inf	-5.31	<0.0001
Eggs laid on 4:1						
	15d - 25d	-0.32	0.33	Inf	-0.97	0.60
	15d - 5d	-0.56	0.32	Inf	-1.77	0.18
	25d - 5d	-0.24	0.30	Inf	-0.82	0.69

Table S18

Table S18: Post-hoc Tukey tests comparing numbers of larvae recovered from each diet patch by parental age group.

	Contrast	Estimate	SE	Z ratio	p value
5d parents					
	1:1 - 1:4	0.50	0.23	2.21	0.07
	1:1 - 4:1	0.36	0.22	1.67	0.22
	1:4 - 4:1	-0.14	0.24	-0.56	0.84
15d parents					
	1:1 - 1:4	1.41	0.26	5.50	<0.0001
	1:1 - 4:1	0.85	0.21	4.04	<0.001
	1:4 - 4:1	-0.56	0.29	-1.95	0.12
25d parents					
	1:1 - 1:4	1.05	0.26	4.03	<0.001
	1:1 - 4:1	0.22	0.20	1.10	0.52
	1:4 - 4:1	-0.83	0.27	-3.08	<0.001

Table S19

Table S19: Post-hoc Tukey tests comparing the probability of eclosion by 14d in larvae reared on the three P:C diet types, separated by parental age group.

	Contrast	Estimate	SE	Z ratio	p value
5d parents					
	1:1 - 1:4	0.50	0.37	1.36	0.36
	1:1 - 4:1	0.01	0.39	0.02	1.0
	1:4 - 4:1	-0.49	0.37	-1.33	0.38
15d parents					
	1:1 - 1:4	-0.99	0.339	-2.90	0.01
	1:1 - 4:1	2.21	0.42	5.28	<0.0001
	1:4 - 4:1	3.20	0.43	7.43	<0.0001

Table S20

Table S20: Post-hoc Tukey tests comparing the probability of eclosion by 14d in larvae of 5d versus 15d parents, separated by rearing diet.

	Contrast	Estimate	SE	Z ratio	p value
1:4 reared					
	15d parents - 5d parents	-0.50	0.34	-1.45	0.15
1:1 reared					
	15d parents - 5d parents	-1.98	0.37	-5.38	<0.0001
4:1 reared					
	15d parents - 5d parents	-4.19	0.46	-9.07	<0.0001

Table S21

Table S21: Post-hoc Tukey tests comparing time to eclosion in days in larvae of 5d versus 15d parents, separated by rearing diet.

	Contrast	Estimate	SE	Z ratio	p value
1:4 reared					
	15d parents - 5d parents	0.22	0.02	12.2	<0.0001
1:1 reared					
	15d parents - 5d parents	0.27	0.02	13.3	<0.0001

Table S22

Table 22: Post-hoc Tukey tests comparing time to eclosion in days in larvae reared on 1:4 or 1:1 P:C diets, separated by parental age group.

	Contrast	Estimate	SE	Z ratio	p value
5d parents					
	1:1 - 1:4	-0.09	0.02	-4.80	<0.0001
15d parents					
	1:1 - 1:4	-0.04	0.02	-1.91	0.06

Table S23

Table S23: Post-hoc Tukey tests comparing wing lengths of male and female offspring with 5d or 15d parents, grouped by rearing diet.

	Contrast	Estimate	SE	Z ratio	p value
Wing length, males					
1:4					
	15d parents - 5d parents	0.12	0.03	3.80	<0.001
1:1					
	15d parents – 5d parents	-0.17	0.03	-5.22	<0.0001
4:1					
	15d parents - 5d parents	0.13	0.04	3.23	0.001
Wing length, females					
1:4					
	15d parents - 5d parents	0.14	0.03	4.20	<0.0001
1:1					
	15d parents - 5d parents	-0.23	0.03	-6.64	<0.0001
4:1					
	15d parents - 5d parents	0.08	0.04	1.75	0.08

Table S24

Table S24: Post-hoc Tukey tests comparing thorax lengths of male and female offspring with 5d or 15d parents, grouped by rearing diet.

	Contrast	Estimate	SE	Z ratio	p value
Thorax length, males					
1:4					
	15d parents - 5d parents	0.06	0.02	2.81	<0.01
1:1					
	15d parents - 5d parents	-0.002	0.02	-0.10	0.92
4:1					
	15d parents - 5d parents	0.05	0.03	1.92	0.06
Thorax length, females					
1:4					
	15d parents - 5d parents	0.02	0.02	1.18	0.24
1:1					
	15d parents - 5d parents	-0.04	0.02	-2.01	0.04
4:1					
	15d parents - 5d parents	-0.03	0.03	-0.98	0.33

Table S25

Table S25: Post-hoc Tukey tests comparing wing length divided by thorax length in male and female offspring with 5d or 15d parents, grouped by rearing diet.

	Contrast	Estimate	SE	Z ratio	p value
Wing/ thorax, males					
1:4					
	15d parents - 5d parents	0.005	0.08	0.06	0.95
1:1					
	15d parents - 5d parents	-0.19	0.09	-2.24	0.03
4:1					
	15d parents - 5d parents	-0.001	0.11	-0.01	0.99
Wing/ thorax, females					
1:4					
	15d parents - 5d parents	0.11	0.06	1.86	0.06
1:1					
	15d parents - 5d parents	-0.15	0.06	-2.33	0.02
4:1					
	15d parents - 5d parents	0.18	0.08	2.24	0.03
